# Polymorphic duplicate genes and persistent non-coding sequences reveal heterogeneous patterns of mitochondrial DNA loss in salamanders

**DOI:** 10.1186/s12864-017-4358-2

**Published:** 2017-12-28

**Authors:** Rebecca A. Chong, Rachel Lockridge Mueller

**Affiliations:** 1 Department of Biology, Colorado State University, Fort Collins, CO 80523-1878 USA; 2 Present address: Department of Biology, University of Hawai’i at Mānoa, Honolulu, HI 96822 USA

**Keywords:** Duplication-random loss model, Gene rearrangement, Pseudogene, Mitochondrial genome evolution, Repetitive DNA, *Aneides*

## Abstract

**Background:**

Mitochondria are the site of the citric acid cycle and oxidative phosphorylation (OXPHOS). In metazoans, the mitochondrial genome is a small, circular molecule averaging 16.5 kb in length. Despite evolutionarily conserved gene content, metazoan mitochondrial genomes show a diversity of gene orders most commonly explained by the duplication-random loss (DRL) model. In the DRL model, (1) a sequence of genes is duplicated in tandem, (2) one paralog sustains a loss-of-function mutation, resulting in selection to retain the other copy, and (3) the non-functional paralog is eventually deleted from the genome. Despite its apparent role in generating mitochondrial gene order diversity, little is known about the tempo and mode of random gene loss after duplication events. Here, we determine mitochondrial gene order across the salamander genus *Aneides*, which was previously shown to include at least two DRL-mediated rearrangement events. We then analyze these gene orders in a phylogenetic context to reveal patterns of DNA loss after mitochondrial gene duplication.

**Results:**

We identified two separate duplication events that resulted in mitochondrial gene rearrangements in *Aneides*; one occurred at the base of the clade tens of millions of years ago, while the other occurred much more recently (i.e. within a single species), resulting in gene order polymorphism and paralogs that are readily identifiable. We demonstrate that near-complete removal of duplicate rRNA genes has occurred since the recent duplication event, whereas duplicate protein-coding genes persist as pseudogenes and duplicate tRNAs persist as functionally intact paralogs. In addition, we show that non-coding DNA duplicated at the base of the clade has persisted across species for tens of millions of years.

**Conclusions:**

The evolutionary history of the mitochondrial genome, from its inception as a bacterial endosymbiont, includes massive genomic reduction. Consistent with this overall trend, selection for efficiency of mitochondrial replication and transcription has been hypothesized to favor elimination of extra sequence. Our results, however, suggest that there may be no strong disadvantage to extraneous sequences in salamander mitochondrial genomes, although duplicate rRNA genes may be deleterious.

**Electronic supplementary material:**

The online version of this article (10.1186/s12864-017-4358-2) contains supplementary material, which is available to authorized users.

## Background

Mitochondria are the site for the citric acid cycle and oxidative phosphorylation (OXPHOS), the final steps of ATP synthesis via cellular respiration. These intracellular organelles have their own genome, which in metazoans is a small, circular molecule averaging 16.5 kb in length [1, 2]. Metazoan mitochondrial genomes typically encode 13 electron transport proteins, two rRNAs, and 22 tRNAs [2]. This gene content is evolutionarily conserved, likely reflecting the fitness advantage of having the proteins involved in primary electron transfer (i.e. redox chemistry) co-localized with the genes that encode them, allowing direct redox control of gene expression [3]. Other hypotheses to explain why this conserved set of genes has remained in a distinct mitochondrial genome, rather than being transferred to the nuclear genome, include (1) their role in redox damage sensing [4], and (2) the hydrophobicity of their protein products, which precludes their import back into the mitochondria [5]. In contrast to such evolutionarily conserved gene content, metazoan mitochondrial genomes show a diversity of gene orders that result from gene rearrangement [1, 6–8]. Many mitochondrial gene orders are hypothesized to reflect rearrangement by neutral processes [9, 10], although tRNA positions within the vertebrate mitochondrial genome have been hypothesized to reflect selection for translational capacity [11].

The most commonly invoked model for mitochondrial gene rearrangement is the duplication-random loss (DRL) model. In the DRL model, a sequence of genes is initially duplicated in tandem. Following this initial duplication, one paralog sustains a loss-of-function mutation, resulting in strong selective pressure to retain the other gene copy functionally intact. The non-functional paralog continues to accumulate mutations and is eventually removed from the genome by deletion. Depending on which gene copy is ultimately deleted, this process may restore the original gene order or result in a novel gene order [12, 13].

The DRL model has been invoked to explain the variation in mitochondrial gene order observed in diverse animal clades including lizards, insects, and caecilians [8, 14–18]. Evidence for the DRL model includes the presence of mitochondrial pseudogenes that can be identified as paralogs of rearranged genes [19]. Despite its apparent role in generating mitochondrial gene order diversity, little is known about the tempo and mode of random gene loss after duplication events. For example, does loss of gene duplicates occur slowly, reflecting rare deletion events and/or their fixation solely by genetic drift? Alternatively, does loss of gene duplicates occur quickly, reflecting frequent deletion events and/or positive selection for deletions?

Selection would play a role in the deletion of duplicate genes if their presence in the genome negatively impacted mitochondrial function. This could occur by increasing the time required to complete transcription of mitochondrial genes and/or replication of the mitochondrial genome [20, 21]. It could also occur by increasing susceptibility to harmful gain-of-function mutations [22] or by shifting genes to a more vulnerable position along the mutation gradient [23]. In such cases, selection would favor the elimination of all extraneous sequence, irrespective of its identity. Alternatively, expression of mitochondrial pseudogenes could result in the production of non-functional proteins [24]. Finally, expression of additional functional gene copies could result in changes to the stoichiometry of mitochondrial proteins [25, 26]. In these latter two cases, selection would favor mutations that delete these specific sequences or otherwise diminish their contribution to the mitochondrial proteome.

Much of our current understanding of mitochondrial gene order variation comes from comparisons across highly divergent clades such as bird families and amphibian orders, where rearrangements have been fixed deep in evolutionary time [1, 19, 27, 28]. In these systems, gene loss occurred far enough in the past that little or no trace of duplicated sequences remain in the genomes of extant taxa; this precludes the study of the tempo and mode of paralog loss. In contrast, the study of more recent gene rearrangements — where duplicates and intermediate stages of loss are polymorphic within species or fixed between closely related lineages — allow the study of random loss “in action.” Such studies remain relatively rare [29, 30].

Salamanders, an amphibian clade of 708 species [31], include both typical vertebrate mitochondrial genomes and a number of independently derived gene rearrangements consistent with origination through the DRL model [19]. Because this mitochondrial genomic diversity is unusual among vertebrates, salamanders are a promising taxon for identifying recent gene rearrangements appropriate for studies of tempo and mode of duplicate gene loss. Within salamanders, the six-species genus *Aneides* includes two species — *A. hardii* and *A. flavipunctatus* — that have distinct, derived mitochondrial gene orders resulting from gene duplications [19, 32]. The duplication in *A. flavipunctatus* encompassed protein coding genes ND6 and cytb, tRNA genes E, T, and P, and an intergenic spacer found in diverse salamander clades [19, 28, 33, 34]. The mitochondrial genome of *A. hardii* shows evidence of having undergone two duplication events. The first duplication involves a nearly identical region of the genome duplicated in *A. flavipunctatus*, suggesting that it may have occurred in the common ancestor of these two species. The second duplication in *A. hardii* involves part of the first duplication as well as the control region, 12S, 16S, ND1, and tRNAs F, V, and L [19].

For this study, we examined mitochondrial gene order in multiple population-level lineages of *Aneides* in a phylogenetic context to pinpoint the evolutionary origins of gene duplications. Our goals were to (1) determine whether the similar duplications in *A. hardii* and *A. flavipunctatus* are, in fact, a synapomorphy for some/all species in the genus, (2) determine whether variation in gene order exists between any closely related lineages or within species, and (3) examine the tempo and mode of duplicate gene loss. We identified two separate duplication events that resulted in mitochondrial gene rearrangements. One such rearrangement occurred at the base of the clade tens of millions of years ago, while the other occurred much more recently (i.e. within a single species), resulting in gene order polymorphism and paralogs that are readily identifiable. We demonstrate that near-complete removal of duplicate rRNA genes has occurred since the recent duplication event, whereas duplicate protein-coding genes persist as pseudogenes and duplicate tRNAs persist as functionally intact paralogs. In addition, we show that non-coding DNA duplicated at the base of the clade has persisted across species for tens of millions of years. Taken together, our results suggest that, although there may be no strong disadvantage to extraneous sequences in salamander mitochondrial genomes, duplicate rRNA genes may be deleterious.

## Methods

### Taxon selection and sampling strategy

Our first objective was to estimate a comprehensive phylogeny for *Aneides* to provide the evolutionary context for analysis of mitochondrial gene rearrangements. To this end, we collected nuclear gene sequence data from four individuals for all six species in the clade (Table 1)*.* We included additional samples of *A. flavipunctatus* because previous studies have documented high levels of divergence across populations in this species [35–37]. We also included an additional sample of *A. hardii* because a previous study identified a novel gene order in this species [19]. We included four related plethodontid salamander species as outgroups (*Ensatina eschscholtzii, Hydromantes brunus, Plethodon elongatus,* and *Desmognathus fuscus*) [32, 38, 39]. All work was completed in compliance with Colorado State University’s Institutional Animal Care and Use Committee (Protocol 08-184A-02).

**Table 1:** Specimen information for individuals included in this study and their voucher numbers, locality information, and GenBank accession numbers for nuclear loci

Species	Voucher	State: County	BDNF	POMC	RAG1	Source
*Aneides aeneus*	DH74978	GA: Chatooga	MF946473	MF946498	MF946523	*This study*
*Aneides aeneus*	DH74985	GA: Chatooga	MF946474	MF946499	MF946524	*This study*
*Aneides aeneus*	DH77583	KY: Letcher	MF946475	MF946500	MF946525	*This study*
*Aneides aeneus*	DH77584	KY: Letcher	MF946476	MF946501	MF946526	*This study*
*Aneides ferreus*	MVZ219942	CA: Siskiyou	MF946477	MF946502	MF946527	*This study*
*Aneides ferreus*	MVZ219953	OR: Douglas	MF946478	MF946503	MF946528	*This study*
*Aneides ferreus*	MVZ219958	OR: Linn	MF946479	MF946504	MF946529	*This study*
*Aneides ferreus*	RCT545	CA: Del Norte	MF946480	MF946505	MF946530	*This study*
*Aneides flavipunctatus*	AGC299	CA: Santa Cruz	MF946481	MF946506	MF946531	*This study*
*Aneides flavipunctatus*	MVZ219973	CA: Siskiyou	EU275895	EU275849	EU275809	Vieites et al. [38]
*Aneides flavipunctatus*	MVZ219977	CA: Sonoma	MF946482	MF946507	MF946532	*This study*
*Aneides flavipunctatus*	RAC080	CA: Mendocino	MF946483	MF946508	MF946533	*This study*
*Aneides flavipunctatus*	RCT481	CA: Shasta	MF946484	MF946509	MF946534	*This study*
*Aneides flavipunctatus*	RLM172	CA: Del Norte	MF946485	MF946510	MF946535	*This study*
*Aneides hardii*	MVZ226110	NM: Otero	EU275857	EU275811	EU275780	Vieites et al. [38]
*Aneides hardii*	RAC020	NM: Lin	MF946486	MF946511	MF946536	*This study*
*Aneides hardii*	RAC025	NM: Otero	MF946487	MF946512	MF946537	*This study*
*Aneides hardii*	RAC042	NM: Lin	MF946488	MF946513	MF946538	*This study*
*Aneides hardii*	RAC054	NM: Lin	MF946489	MF946514	MF946539	*This study*
*Aneides lugubris*	MVZ230722	CA: San Diego	MF946490	MF946515	MF946540	*This study*
*Aneides lugubris*	MVZ249828	CA: Mariposa	MF946491	MF946516	MF946541	*This study*
*Aneides lugubris*	RAC060	CA: Santa Clara	MF946492	MF946517	MF946542	*This study*
*Aneides lugubris*	RAC081	CA: Mendocino	MF946493	MF946518	MF946543	*This study*
*Aneides vagrans*	HBS26688	CA: Mendocino	MF946494	MF946519	MF946544	*This study*
*Aneides vagrans*	MVZ219886	CA: Del Norte	MF946495	MF946520	MF946545	*This study*
*Aneides vagrans*	MVZ220991	CA: Humboldt	MF946496	MF946521	MF946546	*This study*
*Aneides vagrans*	RAC073	CA: Humboldt	MF946497	MF946522	MF946547	*This study*
*Ensatina eschscholtzii*	MVZ236171	CA: San Luis Obispo	EU275862	EU275816	EU275785	Vieites et al. [38]
*Desmognathus fuscus*	MVZ224931	MA: Franklin	EU275858	EU275812	EU275781	Vieites et al. [38]
*Hydromantes brunus*	MVZ238576	CA: Mariposa	EU275871	EU275825	EU275790	Vieites et al. [38]
*Plethodon elengatus*	MVZ220003	CA: Del Norte	EU275882	EU275836	AY650120	Wiens et al. [58]; Vieites et al. [38]

Our second objective was to compare gene order both within and among species of *Aneides* to pinpoint the evolutionary origins of the gene duplications underlying gene rearrangement. To this end, we collected mitochondrial genome sequence data from individuals that represent nine divergent population-level lineages of *Aneides* (Table 2). Wherever possible, we collected mitochondrial genome sequence data from individuals sampled in the phylogenetic analysis to maximize overlap between the two datasets. As above, we sampled multiple individuals from both *A. flavipunctatus* and *A. hardii*.

**Table 2:** Individuals used in this study and their voucher numbers, total number of Illumina MiSeq reads, total number of base pairs, total number of contigs used in mitochondrial genome assembly, and sequence length (kb) of mitochondrial genome annotation

Species	Voucher	Number of reads	Total base pairs	Mitochondrial contigs	Mitochondrial genome annotation (kb)
*A. aeneus*	DH77584	1,648,165	510,564,223	1	16.8
*A. ferreus*	RCT545	1,181,785	365,727,056	5	16.4
*A. flavipunctatus*	MVZ219977	1,165,899	349,989,009	5	17.1
*A. flavipunctatus*	RLM172	1,044,399	308,615,225	4	17.5
*A. flavipunctatus*	AY728214	–	–	1	20.2
*A. hardii*	RAC25	931,319	284,267,433	6	18.7
*A. hardii*	RAC42	1,708,754	507,960,897	5	17.9
*A. hardii*	RAC54	1,280,531	378,635,736	1	17.0
*A. hardii*	AY728226	–	–	1	22.2
*A. lugubris*	MVZ249828	728,185	199,882,489	7	17.0
*A. vagrans*	MVZ220991	832,332	252,211,876	5	16.5

### DNA amplification and sequencing for phylogenetic analyses

For each sample, we extracted total genomic DNA from either flash-frozen or RNAlater-preserved (Qiagen) liver or tail tissue using the Puregene kit (Qiagen) according to the manufacturer’s protocols. We amplified a portion of the nuclear-encoded genes BDNF, POMC, and RAG1 using the Polymerase Chain Reaction (PCR), which produced amplicons of 720 bps, 489 bps, and 837 bps for each gene, respectively. Primers and PCR reaction conditions were obtained from previously published studies [38]. Briefly, approximately 10 ng of genomic DNA was amplified using 0.25 mM dNTPs, 0.5 μM of each primer, 0.5 U *Taq*, 25 μM MgCl_2_, and 10× reaction buffer (no MgCl_2_) in a 12.5 μL reaction volume. Genes were PCR-amplified for 35 cycles (95 °C 45 s, 56–58 °C 1 min, and 72 °C 1 min). All PCR products were electrophoresed on a 1% agarose SB gel stained with Gel Red (Biotium Inc.) and visualized under ultraviolet light. PCR products were purified using ExoSAP-IT (Affymetrix USB products). PCR products were sequenced in both directions by the University of Chicago Comprehensive Cancer Center’s DNA sequencing and genotyping facility using direct double-strand cycle sequencing with Big Dye v3.1 chemistry (Perkin-Elmer) and an ABI 3730 automated sequencer. Contiguous DNA sequences were aligned and edited using Geneious version R7 (Biomatters), and multiple sequence alignments were initially generated using Muscle v.3.6 [40] and subsequently verified by eye. The open reading frames were also verified for all genes using Geneious R7. All sequences are deposited in GenBank (accession numbers MF946473-MF946547).

### Phylogenetic analyses

We estimated phylogenetic relationships within the genus *Aneides* using both Maximum Likelihood (ML) and Bayesian Inference (BI) on the aligned, concatenated nuclear-gene dataset (BDNF, POMC, and RAG1). We evaluated multiple data partitioning strategies in order to incorporate evolutionary information specific to each gene and codon position. We determined three appropriate data partitions using PartitionFinder v.1.1.1 [41]: (1) codon positions 1 and 2 for BDNF, (2) codon positions 1 and 2 for POMC and RAG1 combined, and (3) codon position 3 for all three genes combined. Using the Akaike Information Criterion to select the best model of nucleotide substitution, we determined JC, F81 + I, and GTR + Γ for each partition, respectively.

We conducted partitioned Bayesian analyses in MrBayes v.3.2 [42] for the concatenated nuclear-gene dataset. We conducted two independent searches consisting of three “heated” and one “cold” Markov chain for 10 million generations with every 1000th sample retained. A rate multiplier was used to allow substitution rates to vary among partitions, and default priors were applied to all model parameters. We assessed convergence of the MCMC using several diagnostics. We viewed trace plots of tree -lnL values and other parameters in Tracer v.1.5 [43]. Trees sampled prior to stationarity (i.e. the first 1000 tree samples) were considered as burn in and were discarded. To determine whether the two independent runs converged on similar results, we examined the split standard deviation for -lnL tree values among chains; values <0.01 were taken to indicate convergence. We also used the program Are We There Yet? (AWTY) [44] to compare changes in the posterior probabilities of split frequencies across the independent runs.

Partitioned Maximum Likelihood analyses were conducted using RAxML v.7.2.5 [45] under the GTR + Γ model of nucleotide substitution for all data partitions identified in the Bayesian analysis. Support values for the inferred relationships were obtained from 1000 nonparametric bootstrap pseudoreplicates.

### Mitochondrial genome sequencing and assembly

We obtained cellular DNA shotgun sequence data, which includes both nuclear and mitochondrial DNA, using the Illumina MiSeq sequencing platform for a total of eight individuals representing all six species of *Aneides*, with additional sampling within *A. hardii* and *A. flavipunctatus* (Table 2). Illumina sequencing libraries were produced for each sample using the IntegenX PrepX-DNA 24 library prep kit (IntegenX). Libraries were pooled equimolar for sequencing, allocating $$ \raisebox{1ex}{$1$}\!\left/ \!\raisebox{-1ex}{$3$}\right. $$ of a 2 × 250 cycle MiSeq run (Illumina) for all samples. Library preparation and sequencing were performed by the University of Idaho Institute for Bioinformatics and Evolutionary Studies (IBEST) Genomics Resources Core facility. All sequences are deposited in the GenBank SRA (PRJNA407969). We screened all shotgun reads to eliminate sequencing adapters, identify and remove contaminants, and trim reads based on quality scores using the bioinformatics pipeline “SeqyClean.py” provided by the IBEST Computational Resources Core. We assembled the remaining shotgun reads into contigs using Newbler v.2.6. We also included published shotgun sequence data for an additional sample of *A. flavipunctatus* [46], resulting in shotgun datasets for nine total individuals.

For each of the nine shotgun sequence datasets, we identified contigs of mitochondrial sequences using tBLASTx; the mitochondrial genome reference sequences of *A. hardii* (AY728226) and *A. flavipunctatus* (AY728214) were used as queries to BLAST against the assembled contigs with an e-value cutoff of 1e-5. Depending on the focal individual, complete (or nearly complete) mitochondrial genomes were represented by one to eight contigs. Several genome assemblies included gaps across regions of low/non-existent sequencing coverage. To close these gaps, as well as verify the assembly of multiple contigs, we developed genome-specific primers for PCR amplification and Sanger sequencing. PCR amplification and Sanger sequencing were performed as described above for the nuclear genes used in the phylogenetic analysis.

### Mitochondrial gene order identification

We annotated mitochondrial genome sequences for each individual using Geneious R7. rRNAs and tRNAs were identified based on sequence similarity with published genomes. All 13 protein-coding sequences were verified by eye for appropriate vertebrate mitochondrial open reading frames and stop codons. For each sample, we determined mitochondrial gene order based on our genome annotations and alignment to reference mitochondrial genomes. We also identified pseudogenes based on sequence similarity to inferred functional gene copies as well as position relative to annotated gene sequences.

### Gene loss analysis

Within *A. hardii*, individuals have different mitochondrial genome haplotypes; one haplotype has additional duplicate genes not present in the other. We thus focused on *A. hardii* to study random gene loss “in action.” To this end, we measured (1) substitutions within duplicate gene sequences and their effects on open reading frame, if applicable, and (2) deletions within duplicate sequences. To perform these measures, we created pairwise alignments of pseudogene sequences and their intact paralogs from within the same genome using ClustalW; the intact paralog serves as a proxy for the sequence immediately following duplication. We scaled deletions of duplicate sequences by substitutions, as has been done in other studies, because the limited salamander fossil record precludes precise estimates of absolute rates; salamander divergence date estimates have large 95% confidence intervals [28, 38, 47–49]. More specifically, we estimated the average number of mitochondrial nucleotide substitutions per site (*D*_*XY*_) between the two different *A. hardii* gene orders. We generated multiple sequence alignments including two individuals representing each gene order for each of the protein-coding genes using translational ClustalW alignment [50] and verified open reading frames and stop codons by eye (see Additional file 1). We then estimated *D*_*XY*_ for a concatenated dataset of all 13 mitochondrial protein-coding genes using DnaSP v 5.10.1 [51], which uses the Jukes and Cantor’s model of nucleotide substitution to estimate genetic divergence between populations and is likely sufficient for such shallow divergences.

## Results and discussion

### *Aneides* phylogeny estimated from nuclear genes

**Fig. 1:**
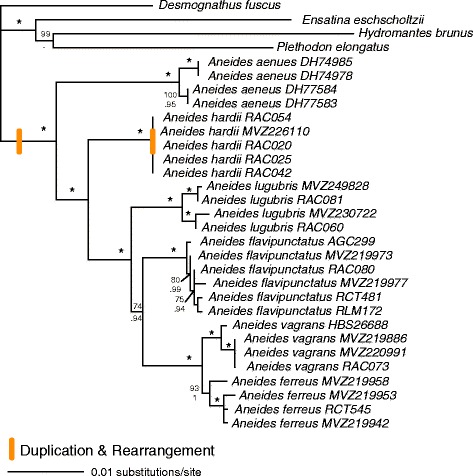
Nuclear phylogeny of *Aneides* with nodes labeled with Maximum Likelihood bootstrap support (MLBP) above and Bayesian posterior probabilities (BPP) below. Both analyses resulted in congruent tree topologies, depicted above, with highly supported nodes (MLBP >99 and BPP > 0.99) denoted with an asterisk, while weakly supported nodes (MLBP <70 or BPP < 0.70) are not labeled. *Aneides hardii* branch lengths are all <0.00001. Orange bars indicate inferred mitochondrial gene duplication and rearrangement events

Despite recent work on salamander phylogenies [38, 39, 52, 53], the phylogenetic relationships within *Aneides* were previously unknown. From our analysis of 2046 bp from three nuclear genes (BDNF, POMC, and RAG1), we estimated a resolved phylogeny for the genus that includes all six species represented by several divergent lineages (Fig. 1). Our results show *A. aeneus* to be sister to the rest of the clade; *A. aeneus* is found in eastern North America, while the remaining five species are only found in western North America. The two Pacific Northwest species — *A. ferreus* and *A. vagrans —* are sister taxa, which together form the clade sister to *A. flavipunctatus. Aneides flavipunctatus* + *A. ferreus + A. vagrans* is sister to *A. lugubris;* these four species are found in the coastal regions of Western North America. *Aneides hardii*, which is restricted to a few isolated mountaintops in southern New Mexico, is sister to the coastal clade. Our results are consistent with the relationships suggested by other studies that included some species of *Aneides* [32, 38, 39, 53].

### Mitochondrial gene order

We identified mitochondrial gene orders for nine *Aneides* samples using shotgun sequence data. The total amount of sequence and average read length varied among samples, but overall, the datasets represent ~ 0.5–1.5% of the nuclear genomes at 1× coverage (Table 2). We produced nearly complete mitochondrial genome sequences from a single contig for two samples. For each of the remaining samples, we produced four to eight contigs that represent large fractions of the mitochondrial genome. We eliminated several assembly gaps using PCR; however, several regions of the genome remain absent from our assemblies. Specifically, the control region and the region between CYTB and ND6 were difficult to assemble, likely reflecting the highly repetitive nature of these two regions [19]. Despite these gaps, our contigs include all 37 mitochondrial genes for each individual; thus, we were able to infer gene order from all nine individuals with confidence. We combined these data with previously published genomes from two additional *Aneides* individuals (one *A. hardii* and one *A. flavipunctatus*) for a total of 11 focal *Aneides* mitochondrial genome sequences (Table 2).

**Fig. 2:**
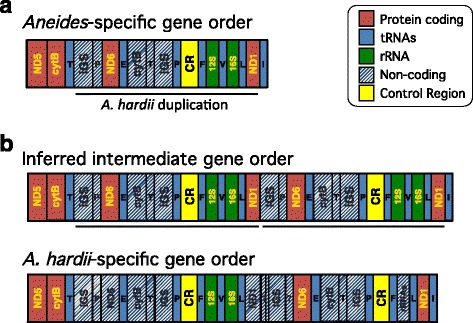
Mitochondrial gene rearrangements in *Aneides hardii*. **a** *Aneides*-specific mitochondrial gene order, which is ancestral for *A. hardii*. The region inferred to have been duplicated in the *A. hardii-*specific duplication event is underlined. **b** Inferred intermediate gene order after *A. hardii*-specific duplication event and the current novel gene order within *A. hardii*

We found evidence of gene rearrangement, relative to the typical vertebrate mitochondrial gene order, in all 11 mitochondrial genome sequences. The mitochondrial gene order in *A. aeneus, A. lugubris, A. vagrans, A. ferreus,* all sampled lineages of *A. flavipunctatus,* and a subset of the sampled lineages of *A. hardii* includes a gene order exchange between ND6 and CYTB as well as the accumulation of repetitive, non-coding DNA in this region (Fig. 2a)*.* This pattern is consistent with (1) an initial duplication of ND6, trn-E, CYTB, trn-T, an intergenic spacer, and trn-P, followed by (2) complete excision of one copy of ND6 + trn-E and decay of the remaining sequences to either recognizable pseudogenes (i.e. ψtrn-P) or unrecognizable tandem repeats [19]. Based on these results, we infer that this gene rearrangement is a synapomorphy for *Aneides* that occurred at the base of the clade (Fig. 1). Divergence date estimates for the split between *Aneides* and its sister taxon (i.e. *Desmognathus + Phaeognathus*) range from 36 to 69 mya, providing maximum and minimum dates for this duplication [38]. Thus, non-coding DNA resulting from the gene duplication at the base of *Aneides* has persisted in the genome of the descendant lineages for tens of millions of years.

### Gene order is polymorphic within *A. hardii*

Of the four new *A. hardii* mitochondrial genomes we sequenced for this study, two individuals have the gene order we show here to be ancestral for *Aneides* (Fig. 2a)*.* The other two individuals have the gene order previously reported for this species that reflects two duplication events [19] (Fig. 2b). Thus, within *A. hardii*, we identified a recent gene rearrangement where duplicates and intermediate stages of paralog loss are polymorphic within the species. Although our sampling found the gene orders in geographically isolated populations, our sample size is too small to infer that the populations are fixed for the difference in gene order.

### Patterns of gene loss following duplication in *A. hardii*

Since the duplication event occurred within the *A. hardii* lineage*,* paralogous sequences within *A. hardii* genomes have begun to diverge from one another, accumulating point substitutions, deletions, and, to a lesser extent, insertions. Here, we summarize patterns of divergence between duplicates for different types of sequences.

#### Protein-coding genes

All of ND6 and at least some portion of ND1 were included in the *A. hardii*-specific duplication (Fig. 2a). The functional copy of ND6 in *A. hardii* is 519 bp long. In contrast, the putative pseudogene copy of ND6 we identified in RAC25 is 521 bp long, reflecting one insertion of a total length of two bp. We were unable to assemble this region of the genome in RAC20. Sequence identity between ND6 and ψND6 is 97%. ψND6 differs from the functional copy by ten point substitutions and two insertions, which result in several amino acid replacements to stop codons. The longest predicted ORF within ψND6 is 139 amino acids long.

The functional copy of ND1 in *A. hardii* is 960 bp. ψND1 is a 676 and 685 bp fragment that shares 91.7% and 94.6% sequence identity with the first 682 bp of the functional ND1 in RAC25 and RAC20, respectively. Specifically, ψND1 differs from the functional copy in RAC20 by 30 point substitutions, a 2 bp deletion, and two insertions (5 bp total), which result in several amino acid replacements to stop codons. Similarly, ψND1 differs from the functional copy in RAC25 by 42 point substitutions, four deletions (11 bp total), and two insertions (5 bp total), resulting in several amino acid replacements to stop codons. The longest predicted ORF within ψND1 is 105 amino acids long in RAC25 and 205 amino acids in RAC20. The ψND1 sequences are 94.7% identical between the two individuals. Because we are unable to confirm the total amount of ND1 that was included in the initial duplication, we are unable to determine the total amount of deleted sequence in ψND1. If ND1 were only partially duplicated, it would have become a pseudogene immediately because of its shortened length.

#### rRNA genes

Both ribosomal RNA genes were included in the *A. hardii*–specific duplication. Duplicate copies of both rRNAs, as well as the intervening tRNA-V, have been almost completely deleted from genomes with the *A. hardii*-specific duplication. The functional copies of these three genes together total 2520 bp of contiguous sequence; however, only a 111-bp-long segment of unrecognizable sequence remains of the duplicates.

#### tRNA genes

tRNA-E, -P, -F, -V, and -L were included in the *A. hardii*-specific duplication. With the exception of one tRNA, we were able to identify all *A. hardii*-specific duplicated tRNA gene copies (except tRNA-V, located between the two rRNAs), which appear to largely remain functional based on intact stem-loop structures and the anticodon sequences. Both individuals with the *A. hardii*-specific duplication retain identical copies of tRNA-P and likely tRNA-E as well (although we were unable to identify a second copy in RAC20). tRNA-L paralog sequences differed by two single-basepair substitutions (one shared, one unique) in both individuals and share 97.7% sequence identity, even though each tRNA sequence is unique. For individuals with the *A. hardii*-specific duplication, tRNA-F sequences upstream of the intact rRNA genes are identical, while tRNA-F sequences adjacent to the pseudogenized rRNA genes are more variable and likely nonfunctional. The latter tRNA-F differs from the former by three bases (in RAC20) and five bases (in RAC25), with only two differences being shared by both individuals. The relatively short length of tRNA genes (~70 bp) limits the likelihood of accumulating substitutions and indels within these genes, which would allow for the conservation and functional persistence of tRNAs over other duplicated genes of greater length.

#### Non-coding sequences

Two regions of non-coding, repetitive DNA remain in all *Aneides* mitochondrial genomes from the ancestral duplication at the base of the clade. These regions are predicted to be the remnants of (1) a copy of CYTB + tRNA-T + IGS and (2) a copy of IGS + tRNA-P. Both were included in the *A. hardii*-specific duplication, and at least a portion of both sequences is retained in duplicate following the *A. hardii* duplication. We were unable to fully resolve these regions of the genome because they are repetitive and could not be assembled with confidence. Average sequencing read depth of these regions is at least double that of the rest of the genome, suggesting that these regions occur at least twice as often.

Taken together, our results show that, following the *A. hardii*-specific duplication event, different duplicate sequences have had dramatically different fates. Ribosomal gene copies have been almost completely deleted from the genome, and the sequence remaining bears no identity to the functional rRNA copies. In contrast, duplicate protein-coding sequences have not been deleted; specifically, ψND6 is now longer than the functional paralog, and ψND1 does not show evidence of substantial deletion. Both duplicated protein-coding sequences have decayed to pseudogenes and now encode short, presumably non-functional ORFs. Duplicate tRNAs (except trn-V) also show no evidence of deletion; tRNA-E, -P, and -L remain apparently functional, retaining 98–100% sequence similarity between the two paralogs.

### Loss of duplicate sequences in *A. hardii* occurred over a shallow evolutionary timescale

We measured the number of substitutions between the *A. hardii* mitochondrial genomes that (1) retained the ancestral *Aneides* gene order, and (2) underwent the *A. hardii*-specific duplication. The average number of nucleotide substitutions per site between the two different mitochondrial gene orders found within *A. hardii* was low (*D*_*XY*_ = 0.024). This result is congruent with our nuclear gene phylogenetic analysis, which shows very low genetic divergence among *A. hardii* individuals (Fig. 1). Thus, all of the duplicate gene sequence decay/loss we identify in the *A. hardii*-specific duplication has occurred in the time taken to accumulate only 2.4% sequence divergence from the ancestral *Aneides* mitochondrial genomes.

### What evolutionary forces may have shaped duplicate gene loss in *A. hardii*?

We have demonstrated that two mitochondrial gene duplication events occurred during the history of the genus *Aneides* — one tens of millions of years ago, and another much more recently within a single species. We have shown that different duplicate sequences have had very different fates: ribosomal gene copies have been almost completely deleted from rearranged *Aneides hardii* genomes, whereas non-coding sequence from the duplication event that occurred at the base of the clade persists in several species of *Aneides.* What do these patterns of paralogous sequence evolution suggest about the evolutionary forces that shape duplicate gene loss in mitochondria?

The evolutionary history of the mitochondrial genome, from its inception as a bacterial endosymbiont to its emergence as an essential organellar genome, includes massive genomic reduction [54–56]. Consistent with this overall trend, it has been hypothesized that selection for efficiency of mitochondrial replication and transcription would favor the elimination of extraneous sequence. Additionally, the accumulation of noncoding DNA can pose a mutational hazard [3–5, 57]. In *Aneides*, we do not see evidence that deletions of extraneous sequence are frequent targets of strong positive selection. Rather, we see (1) non-coding sequences persisting/expanding in length in all species of *Aneides* that originated from a duplication event tens of millions of years ago, (2) non-coding sequences (i.e. repeat sequences and pseudo-protein-coding genes) persisting in the *A. hardii* genomes that underwent a duplication event much more recently, and (3) full-length duplicate tRNAs persisting in *A. hardii* genomes, all of which suggest that these genomes do not rapidly purge duplicated regions. These patterns have at least two possible (somewhat overlapping) interpretations. First, it is possible that duplicate sequences do not strongly negatively impact mitochondrial function through their effects on rates of duplication and transcription or their mutational hazard. Accordingly, insertions and deletions in these sequences reflect the underlying mutational spectrum, and large deletions are relatively rare. Second, it is possible that large deletions may have enough of an impact on duplication/transcription/harmful mutation rate to be targets of positive selection, but that such large deletions are sufficiently rare, relative to substitutions and smaller insertions and deletions, that duplicate sequences persist in *Aneides* genomes, despite some negative fitness consequences.

In contrast to the persistence of duplicate tRNAs and pseudogenized protein-coding genes, as well as the persistence/expansion of repetitive sequence, duplicate ribosomal RNA genes have been almost completely deleted since the recent *A. hardii* duplication event. This suggests that selection may have favored deletion of these sequences. Although the presence of additional identical rRNA gene copies in the mitochondrial genome may be unlikely to have deleterious fitness consequences, the presence of pseudo-rRNA genes that compromise protein translation could negatively impact fitness. Thus, we hypothesize that (1) duplicate rRNA genes sustained mutations that compromised translational capacity, and (2) deletions eliminating such sequences were then favored by selection. With our data, however, we cannot exclude the alternate possibilities that a large deletion eliminating the duplicate rRNA genes was fixed by genetic drift or by selection acting on duplication/transcription. Characterizing the evolutionary fate of other recent rRNA gene duplications would aid in discriminating among these hypotheses.

## Conclusions

Although metazoan mitochondrial genomes have conserved gene content, gene order can vary across taxa, reflecting partial genome duplication and subsequent paralog loss. We identified two duplication events in the history of the salamander genus *Aneides*: one that occurred tens of millions of years ago at the base of the clade, and the other that occurred recently within a single species (*A. hardii*). Our results suggest that some extraneous sequences have remained in *Aneides* mitochondrial genomes for tens of millions of years, while duplicate rRNA genes have been eliminated over much shorter timescales. Together with previous work showing that (1) increased metabolic demand is not correlated with mitochondrial genome streamlining in amphibians [21], and (2) genome expansion does not place genes in more vulnerable positions along a strong mutation gradient in salamanders [23], our results suggest that overall DNA loss is not likely to be a strong target of selection in salamander mitochondrial genomes. This parallels the pattern seen in salamander nuclear genomes, which also show slow rates of DNA loss [47]. Further studies identifying recent rRNA duplications in other taxa are required to test whether additional rRNA genes are functionally disadvantageous and targeted by purifying selection.

## Additional file

Additional file 1: ClustalW-created multiple sequence alignments for each of the mitochondrial protein-coding genes including two individuals representing each *A. hardii* gene order. (TXT 45 kb)
